# Efficient Gene Silencing Mediated by Tobacco Rattle Virus in an Emerging Model Plant *Physalis*

**DOI:** 10.1371/journal.pone.0085534

**Published:** 2014-01-14

**Authors:** Ji-Si Zhang, Jing Zhao, Shaohua Zhang, Chaoying He

**Affiliations:** 1 State Key Laboratory of Systematic and Evolutionary Botany, Institute of Botany, Chinese Academy of Sciences, Beijing, China; 2 University of Chinese Academy of Sciences, Beijing, China; CNRS UMR7622 & University Paris 6 Pierre-et-Marie-Curie, France

## Abstract

The fruit of *Physalis* has a berry and a novelty called inflated calyx syndrome (ICS, also named the ‘Chinese lantern’). Elucidation of the underlying developmental mechanisms of fruit diversity demands an efficient gene functional inference platform. Here, we tested the application of the tobacco rattle virus (TRV)-mediated gene-silencing system in *Physalis floridana*. First, we characterized the putative gene of a phytoene desaturase in *P. floridana* (*PfPDS*). Infecting the leaves of the *Physalis* seedlings with the *PfPDS*-*TRV* vector resulted in a bleached plant, including the developing leaves, floral organs, ICS, berry, and seed. These results indicated that a local VIGS treatment can efficiently induce a systemic mutated phenotype. qRT-PCR analyses revealed that the bleaching extent correlated to the mRNA reduction of the endogenous *PfPDS*. Detailed comparisons of multiple infiltration and growth protocols allowed us to determine the optimal methodologies for VIGS manipulation in *Physalis*. We subsequently utilized this optimized VIGS methodology to downregulate the expression of two MADS-box genes, *MPF2* and *MPF3*, and compared the resulting effects with gene-downregulation mediated by RNA interference (RNAi) methods. The VIGS-mediated gene knockdown plants were found to resemble the mutated phenotypes of floral calyx, fruiting calyx and pollen maturation of the RNAi transgenic plants for both *MPF2* and *MPF3*. Moreover, the two MADS-box genes were appeared to have a novel role in the pedicel development in *P. floridana*. The major advantage of VIGS-based gene knockdown lies in practical aspects of saving time and easy manipulation as compared to the RNAi. Despite the lack of heritability and mosaic mutation phenotypes observed in some organs, the TRV-mediated gene silencing system provides an alternative efficient way to infer gene function in various developmental processes in *Physalis*, thus facilitating understanding of the genetic basis of the evolution and development of the morphological diversities within the Solanaceae.

## Introduction

*Physalis* belongs to the Solanaceae family. In this genus, the calyx enlarges rapidly into a Chinese lantern structure as the berry develops. This enlarged calyx, also called inflated calyx syndrome (ICS), ultimately encloses the entire berry. This ICS trait is a post-floral novelty within the Solanaceae. The origin of this morphological novelty presents intriguing evolutionary questions. We previously demonstrated that *MPF2*, which encodes a MADS-box transcription factor, controls ICS development and male fertility in *Physalis* [1]. Further comparative analyses revealed that heterotopic expression of an *MPF2*-like gene is a key to origin of the novel ICS trait within the Solanaceae [1]–[3]. Sequence divergence of MPF2-like proteins leading to alterations in their interacting proteins is associated with the evolution of ICS [3], [4]. The development of the trait also requires a fertilization signal, and plant hormones like cytokinin and gibberellin are known to be able to replace this signal and trigger ICS development [1], [5]. Moreover, berries of some *Physalis* species, including the vegetable crop *Physalis philadelphica* (tomatillo) as well as *Physalis pubescens* have variable sized berries [6]. Some differentially expressed genes between tomatillo accessions with large and small berries were isolated, and their expression in response to developmental cues might explain their potential roles in berry development and size control [6]. However, the function of these genes in the natural variation of berry size remains elusive. *Physalis* is thus emerging as a model plant in evolutionary biology for the origin of the morphological novelties and natural variation of the complex traits [6]. Nevertheless, the elucidation of the complete genetic architecture responsible for ICS and berry size is still challenging, owing to many technological restrictions.

Mutagenesis-mapping and QTL mapping have enabled great progress in gene functional inference in many species [7]–[9]. However, these strategies cannot be easily applied in *Physalis*. Most species in *Physalis* are self-incompatible, and inter-species crossing is extremely difficult [10]. Gamma-radiation mutagenesis was successfully performed in *Physalis floridana* and plenty of evolutionarily informative mutants were isolated [11]. However, molecular characterization of these mutants is technologically difficult. *P. floridana* is a self-compatible species yet the natural variation in the species is quite low. As such, it is very hard to establish effective mapping populations. Fortunately, reverse genetics analyses using transgenic methodologies are feasible in *Physalis*. Gene silencing through RNA interference (RNAi) has been demonstrated [1], [12], [13]. Nonetheless, these methods depend on *Agrobacterium*-mediated transformation and regeneration in strict aseptic conditions. Moreover, large amounts of labor are required, and the transformation efficiency and regeneration success rates are major limiting factors with RNAi methods in *Physalis*. It often takes 3–5 years for a professional scientist to complete the functional inference for a single *Physalis* gene. All of these factors are currently hampering our progress towards understanding the genetic basis of ICS and berry development. We need alternative efficient experimental methodologies to facilitate the functional analyses of *Physalis* genes.

Virus-induced gene silencing (VIGS) has been an important experimental approach for some time [14]. The RNA degradation mechanism in VIGS is similar to that of the degradation pathways in RNAi [15]–[17]. Compared to RNAi, the greatest advantages of VIGS are that it is more convenient and that it saves time. To date, a variety of virus carriers have been successfully developed [18]–[25]. Many of these viruses are limited to several specific species due to host range and/or meristem exclusion [14]. Tobacco rattle virus (TRV)-mediated gene silencing systems have overcome many of these limitations and have become the most extensively used VIGS system [21], [23]. TRV-VIGS has been efficaciously employed in a number of species in the Solanaceae [22], [23], [26]–[29] but not in *Physalis* species. Nonetheless, the TRV system has also been successfully applied in non-Solanaceous species such as *Arabidopsis thaliana* [21], [30] and the lower eudicots *Papaver somniferum* [31] and *Aquilegia vulgaris* [32]. These previous successes have indicated a broad-spectrum of hosts for TRV methods. Very recently, we have successfully applied this technology in *P. floridana* to address genetic interaction of MADS-box genes *MPF3* and *MPF2* in the development of both flower and ICS [13].

In this work, we optimized the operating protocol for the TRV-VIGS technique in *Physalis*. For this purpose, we first characterized a gene encoding phytoene desaturase in *P. floridana* (*PfPDS*). Gene silencing of *PfPDS* using the TRV vector system resulted in a bleached plant. Bleaching was obvious in leaves, floral organs, ICS, berries, and seeds, thus indicating the applicability of the TRV-mediated VIGS in *Physalis*. The established optimal protocol of TRV-VIGS will facilitate inference of gene function in various developmental processes in *Physalis*. Further exploiting the practical advantages of the VIGS system in *Physalis*, we focused on comparing the resulting effects between VIGS and RNAi in the two regulatory genes *MPF2* and *MPF3*. The VIGS-infected flowers have similar phenotypes compared with their RNAi functional validation results [1], [13], and the VIGS operation needs less time and less labors as compared with the RNAi technology, thus illustrating the advantages of the use of this technique in *Physalis.*

## Materials and Methods

### Plant Material and Growth Conditions

*Physalis floridana* syn. *P. pubescens* [1] was grown in a growth chamber under long-day conditions (8 hrs dark/16 hrs light) with temperature control of 21–25°C. Two-week old *Physalis* seedlings were infected with *PfPDS*-*TRV2* mediated by *Agrobacterium*, either through vacuum infiltration or injection. 20 injection-treated seedlings were cultivated in a glasshouse with a temperature of 26–30°C (Summer in Beijing) as controls.

### Isolation of *PfPDS* in *P. floridana* and RT-PCR Analyses

Total RNA was extracted from leaves, floral buds, all floral organs, and fruits using the Trizol reagent (Invitrogen). After treatment with DNase (TaKaRa), the first strand of cDNA was synthesized with an oligo (dT)_17_ primer using M-MLV Reverse Transcriptase™ (Invitrogen). The full length sequence of the *PfPDS* gene was generated after 5′ and 3′ rapid amplification of cDNA ends (RACE). The PCR products were purified with the High Pure PCR Product Purification Kit (Roche). The primers for 3′RACE were synthesized according to the conserved regions of *PDS* genes from *S. lycopersicum* and *N. benthamiana*. The primers for 5′RACE were designed based on the fragment obtained in the 3′RACE results (Table S1 in File S1). Semi-quantitative RT-PCR analyses were performed and 25 cycles were amplified for both *PFACTIN* and *PfPDS*. The *PFACTIN* gene was used as an internal control. The primers used in this study are presented in Table S1 in File S1.

### Generation of *TRV2* Constructs

A *PfPDS* cDNA fragment was designed for its silencing according to the *PDS*-VIGS used in *S. lycopersicum* [22]. The fragments for *MPF2*-RNAi [1] and for *MPF3*-RNAi [13] were also used to construct *TRV2* vectors. Specific primers were designed for appending *Nco* I and *BamH* I restriction sites (Table S1 in File S1). The amplified products and *TRV2* were double digested with *Nco* I and *BamH* I (TaKaRa), and were then ligated using T_4_ DNA ligase (TaKaRa) to generate *PfPDS*-*TRV2*, *MPF2*-*TRV2*, and *MPF3*-*TRV2*. The ligation products were transformed into *E. coli* and selected on LB medium containing 50 µg/mL kanamycin. Positive clones were screened via PCR using primers 156F and 156R [32].

### Transformation of the *TRV2* Construct into *Agrobacterium*

The positive recombined constructs (*PfPDS*-*TRV2*, *MPF2*-*TRV2*, and *MPF3*-*TRV2*) and plasmid construct (*TRV1*) were extracted by SDS Alkaline Lyses and transformed into competent cells of *Agrobacterium* (strain GV3101) using an Electroporator 2510 (Eppendorf). After 2 hrs of incubation at 28°C, the cells were plated on selective YEB media containing 50 µg/mL kanamycin, 100 µg/mL gentamicin and 25 µg/mL rifampicin for 2 days at 28°C. Colony PCR was performed to further validate the positive clones using primers 156F and 156R [32].

### Preparation of *Agrobacterium* Culture

An individual positive clone was picked and cultivated with vigorous shaking (250 rpm) in 5 mL selective liquid YEB (50 µg/mL kanamycin, 100 µg/mL gentamicin and 25 µg/mL rifampicin) at 28°C until the culture reached an OD_600_ = 2.0. The cultures were inoculated into 250 mL of the selective liquid YEB with 2.5 mL 1.0 M MES and 50 µL 0.1 M acetosyringone added, and shaken vigorously (250 rpm) overnight (12–16 hrs). Cells were collected at 4000 g for 15 mins at 4°C and resuspended in infection buffer (1.0 M MES 10 µL/mL, 1.0 M MgCl_2_ 10 µL/mL, and 0.1 M acetosyringone 2 µL/mL). The cell suspension was incubated at room temperature for at least 3 hrs prior to performing the infections.

### *TRV2* Infection in *P. floridana*

Equal volumes of cell suspensions harboring *TRV1* and *PfPDS*-*TRV2*, *MPF2*-*TRV2*, and *MPF3*-*TRV2*, respectively, were mixed with addition of Silwet (GE Healthcare) to a concentration of 5 µL/100 mL for agro-infiltration via vacuum or injection. 200 mL mixed solution of *TRV1* and *TRV2* suspensions was used to vacuum infiltrate or inject about 60 seedlings. In vacuum infiltration, the seedlings were divided into either the whole plant group or the aerial organ-treated group. For each group, the vacuum time lasted for either 2 or 5 mins. After treatment, the seedlings were drained and transplanted to fresh soil. The leaves of the seedlings were treated by injection using sterile 5 mL disposable syringes without needles (ZhiYu). All of the infected seedlings were cultivated in darkness for 48 hrs and then shifted into a growth chamber or a glasshouse with the temperature of 21–25°C under long-day conditions.

### qRT-PCR Assays

The qRT-PCR analyses were performed using the SYBR *Premix Ex Taq*™ (Perfect Real Time) Kit (TaKaRa) according to the manufacturer’s instructions using an Mx3000P (Stratagene) real-time system. The procedure of amplification was a custom two-step program, which had 1 cycle of melting for 30 sec at 95°C, and 40 cycles of melting for 5 sec at 95°C and then annealing for 45 sec at 60°C. The primers used in this study are presented in Table S1 in File S1.

### Morphological Analyses

The phenotypic variations observed were photographed using a Nikon camera attached to a stereomicroscope. Berry weight was measured and seed number per berry was counted. Seed germination was evaluated in a growth chamber with temperature of 21–25°C under long-day conditions. For scanning electronic microscopy (SEM), fresh material was fixed in formalin-acetic acid-alcohol solution, sputter-coated with gold, and examined with a digital scanning microscope (Hitachi S-800, Japan). Calyx, pedicel, and cell size were quantified using the AxioVision LE image-processing program (http://www.zeiss.de). Pollen maturation was evaluated using iodine-potassium iodide (I_2_-KI) staining. All *p* values are based on the two-tailed Student’s *t*-tests.

### Sequencing and Phylogenetic Analyses

Sequencing was performed by BGI (Beijing Genomics Institute). Sequence identity analysis was performed using the NCBI BLAST algorithm suite (http://blast.ncbi.nlm.nih.gov). The sequences of functionally characterized *PDS* genes were aligned using Clustalx 1.83 [33]. The Neighbor-joining tree was generated by MEGA 5.0 with 1000 bootstraps replicates [34]. The nucleotide sequence of *PfPDS* reported in this paper has been deposited in the NCBI database under accession number of JX255734.

## Results and Discussion

Molecular Characterization of the Phytoene Desaturase Gene in *P. floridana* Phytoene desaturase, a key enzyme in the carotenoid biosynthetic pathway, is often chosen for use as a reporter in gene silencing systems such as virus-induced gene silencing (VIGS). The phytoene desaturase gene (*PDS*) has one copy in most species examined, and has a wide expression domain [21], [22], [30]. Once *PDS* is silenced, the plant no longer has a functional carotenoid biosynthetic pathway, thus resulting in bleached organ phenotypes [35]. Bleached phenotypes are easy to observe, and this is the primary reason that *PDS* is used as a reporter for the successful application of VIGS.

In order to establish VIGS in *Physalis*, the *PDS* gene from *Physalis floridana* (*PfPDS*) was isolated and cloned. Specific primers were designed according to the conserved region of the *PDS* genes in *Solanum lycopersicum* (EF650011), *Nicotiana benthamiana* (EU165355), and *Nicotiana tabacum* (AJ616742). After 5′- and 3′-RACE, a 2044-bp long cDNA fragment was obtained from *P. floridana*. It putatively encoded a protein consisting of 582 amino acids, sharing 95% and 91% sequence identities with SlyPDS from *S. lycopersicum* and NbePDS from *N. benthamiana*, respectively. The obtained cDNA fragment from *Physalis* was designated as *PfPDS*. A Neighbor-Joining phylogenetic tree shows a close relationship of PfPDS with the functionally characterized PDS enzymes (Figure S1A in File S1), thus indicating that *PfPDS* is a *PDS* ortholog.

We also investigated the expression pattern of *PfPDS*. Total RNA from leaves, floral buds, all floral organs and fruits were subjected to RT-PCR analyses. The gene is apparently highly expressed in all of the organs that we tested (Figure S1B in File S1). As with the *PDS* genes from various plant species [21], [22], [30], *PfPDS* is constitutively expressed in different organs with little fluctuation in expression levels. Therefore, knocking *PfPDS* down in *Physalis* was expected to cause a bleaching phenotype in these organs.

### Construction of *TRV2* Vectors

**Figure 1:**
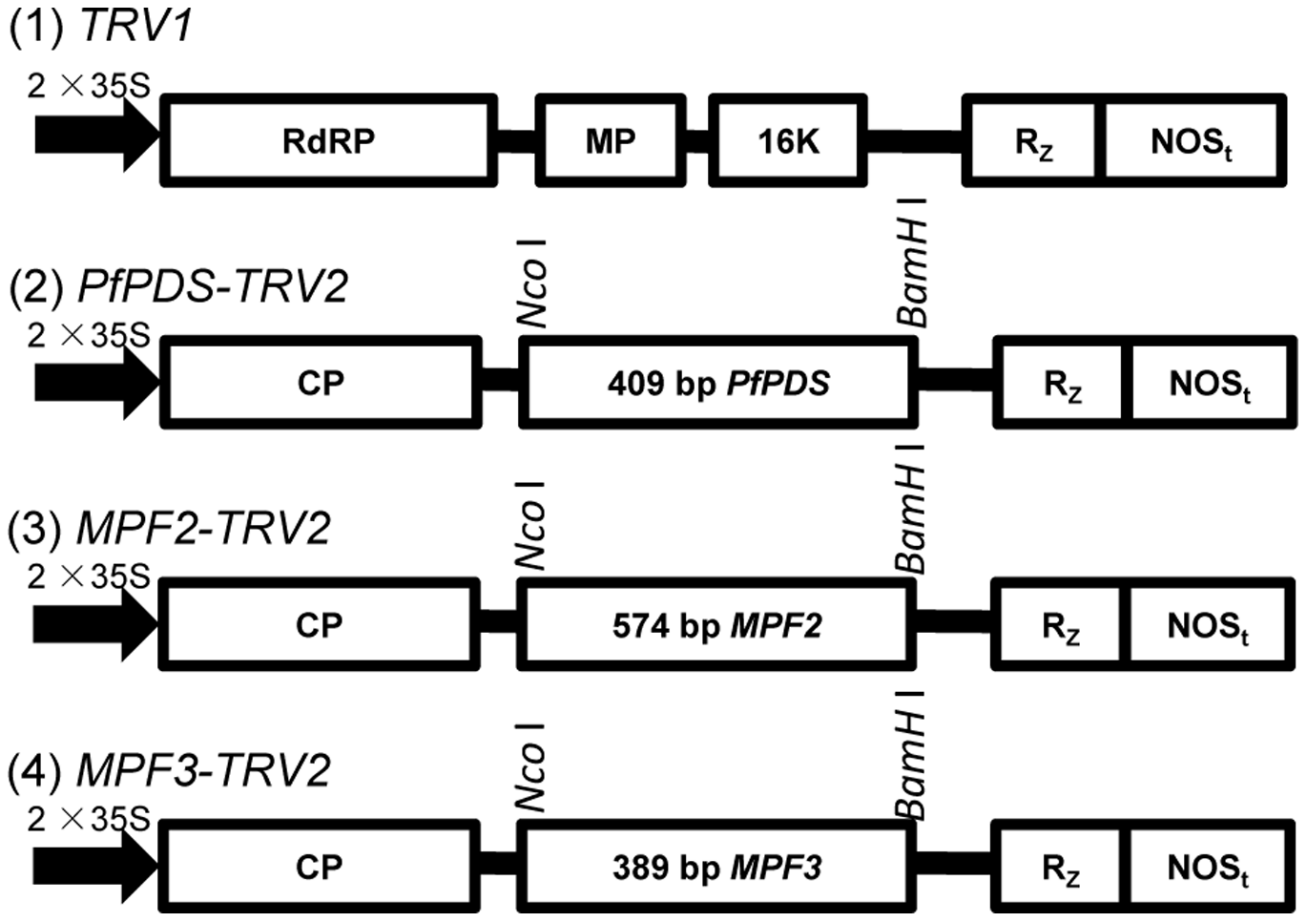
Organization of TRV-VIGS vectors. *TRV* cDNA clones were placed in between duplicated CaMV 35S promoters (2×35 S) and the nopaline synthase terminator (NOSt) in a T-DNA vector [22]. RdRp: RNA-dependant RNA polymerase; MP: movement protein; 16 K: cysteine rich protein; Rz: self-cleaving ribozyme; CP: coat protein. *PfPDS-*, *MPF2-,* and *MPF3*-specific fragments were inserted separately into *TRV*2 using *Nco* I and *BamH* I restriction sites.

Since the TRV carrier seems to have a broad host-range compared to other carriers, we decided to apply this system in *Physalis*. The improved TRV vector consists of two kinds of virion: TRV1 and TRV2 [22], [23]. *TRV1* is responsible for encoding a 29 kDa movement protein and a 16 kDa cysteine-rich protein, which can duplicate and move independently without TRV2. However, TRV2, which is a coat protein and used for the expression of target gene RNA, needs TRV1 to implement the transfer in the host. We chose a 409-bp fragment of *PfPDS* as the insert region and generated the *PfPDS*-*TRV2* construct. We also generated constructs for two MADS-box genes: a 574-bp fragment of *MPF2*-RNAi [1] for the *MPF2*-*TRV2* construct, and a 389-bp fragment of *MPF3*-RNAi [13] for the *MPF3*-*TRV2* construct (Figure 1). The selected fragment of *PfPDS* is the counterpart to the *PDS* fragments used in the previous VIGS analyses [22]. The selection of this *PfPDS* fragment would theoretically result in the successful silencing of the target gene in the corresponding constructed TRV2 infected *Physalis*.

### Infection Efficiency of Different Approaches under Different Temperature Conditions

Lessons from previous VIGS practices and reports suggest that manipulation of both the infection protocols as well as the plant growth conditions is critical for the successful establishment of VIGS in a new host system. Several infection protocols have been examined, and include toothpick inoculation [36], injection infiltration [29], high pressure injection [22], and vacuum infiltration [37]. Not surprisingly, the efficacy of each infection protocol is inconsistent among various plant species and viral vectors. Very recently, the TRV gene silencing system was shown to yield a high infection efficiency using injection protocols in both *N. benthamiana* and *S. lycopersicum* [38].

In order to find a suitable way to infect *P. floridana* with TRV, we compared the survival rate and infection efficiency of *PfPDS*-*TRV* plants using either vacuum infiltration or injection. In the vacuum infiltration experiments, either the whole seedling or the aerial organs were held continuously under vacuum for either 2 or 5 mins, thus dividing the samples into four treatment groups (Table 1). 20 seedlings were included in each vacuum group. Eighty-one seedlings were used in local (leaf) injection experiments, and divided into 2 further groups according to the temperature (Table 1). After treatments, the seedlings were transplanted into pots. For the vacuum infiltration groups, all treated seedlings looked filmy and wilted. The seedlings treated using injection seemed to grow normally. These treated plants were immediately incubated in darkness for 48 hrs, and then cultivated under long-day conditions. All seedlings in the vacuum infiltration groups died, while all 81 seedlings in injection groups survived. The zero-survival rate in the vacuumed groups suggested that the wounding resulting from vacuum infiltration was too severe to enable recovery. In the injection groups, on the seventh day, the emerging leaves of some of the plants showed bleaching. By the tenth day, 47 plants had developed leaves with some bleaching. Therefore, injection was the best method of infection of *Physalis* that we tried, and it seemed to have a minor impact on post-treatment seedling growth.

**Table 1:** Seedling survival and infected efficiency in different treatments.

Infectionpattern	Infectiontissue	Treatedtime (min)	SeedlingsTreated	Temperature (°C)	Seedlingsurvived	Seedlings withsyndrome	Infectionefficiency
Vacuum infiltration	Whole-plant	2	20	21–25	0	/	/
		5	20	21–25	0	/	/
	Aerial tissues	2	20	21–25	0	/	/
		5	20	21–25	0	/	/
Injection	Leaves	/	61	21–25	61	47	77%
			20	26–30	20	0	0

The efficacy of VIGS seemed to be temperature-sensitive (Table 1). The 61 injected plants were cultivated under 21–25°C temperatures and 77% of the plants featured bleaching phenotypes, while in the 20 lines that were cultivated in a greenhouse with a temperature ranging between 26–30°C, we did not see the bleaching phenotype in any of the plants. These observations suggest that the optimal temperature for successful use of the TRV system for gene silencing in *Physalis* is 21–25°C.

For VIGS systems in other species, it has been demonstrated that bleaching phenotypes can appear continuously on the newly developing organs for as long as three months in many cases, and that plants can gradually recover and return to normal pigmentation patterns [21], [39]. In *Physalis*, a similar phenomenon was observed. The occurrence of the bleaching phenotype in *Physalis* lasted for three months, and then the phenotype gradually changed to resemble that of the wild-type. This restoration of the wild-type pigmentation was even observed for VIGS-treated plants grown under the optimal growth conditions. The reason for this return to normal pigmentation remains unclear.

Nonetheless, our results suggest that TRV-mediated gene silencing is transient but applicable in *Physalis*. Further, our results showed that injection is an efficacious method of infiltration, and that the optimal temperature range for VIGS in *Physalis* is between 21–25°C.

### Local *PfPDS*-*TRV2* Infection Induces Systemic Bleaching Phenotypes

**Figure 2:**
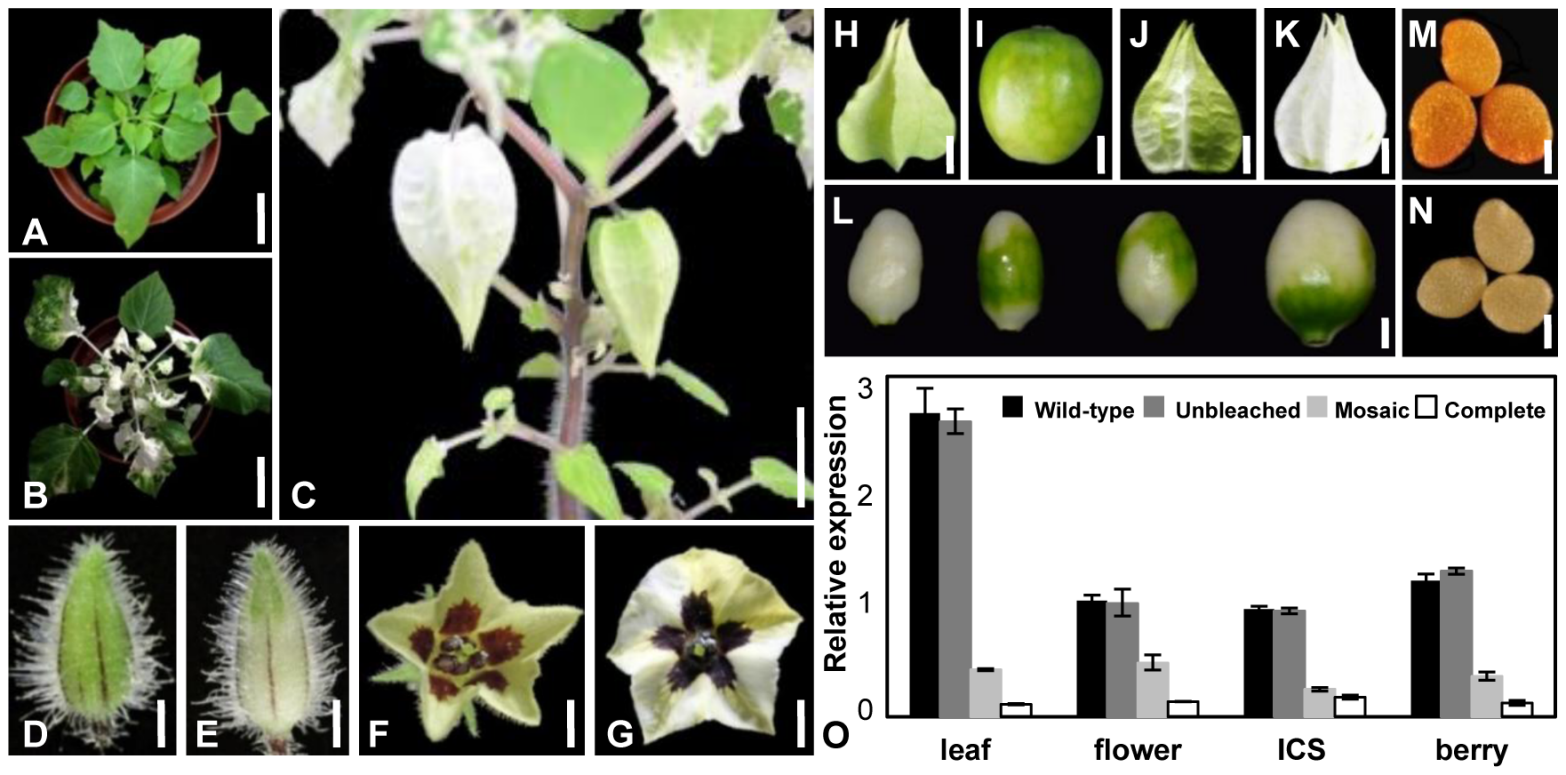
Local treatments induce a systemic syndrome. (**A**) A wild-type seedling of *P. floridana*. (**B**) Phenotypic variation in a seedling after *PfPDS*-*TRV2* infection for one week. Bars = 5.0 cm. (**C**) A 3-month old plant infected with *PfPDS*-*TRV2*. Bars = 1.5 cm. (**D–G**) Floral phenotypic variations. In comparison with wild-type floral bud (**D**) and mature flower (**F**), the floral bud (**E**) and mature flower (**G**) from the *PfPDS*-*TRV2* infected plants are bleached. Bars = 10 mm in **D** and **E**, and 25 mm in **F** and **G**. (**H**) ICS from wild-type *Physalis*. (**I**) Berry from wild-type *Physalis*. (**J**) Mosaic bleached ICS. (**K**) Completely bleached ICS. (**L**) Bleached berries with different bleaching degrees from the *PfPDS*-*TRV2* infected plants. Bars = 50 mm in **H**, **J** and **K**, and 25 mm in **I** and **L**. (**M**) Wild-type seeds. (**N**) Bleached seeds. Bars = 5 mm. (**O**) Expressions of *PfPDS* in the *PfPDS*-*TRV2* infected plants. The black column is for the wild type and the other columns are for the *PfPDS*-*TRV2* infected plants with different degrees of bleaching. qRT-PCR was performed using total RNA from the organs indicated. *PFACTIN* was used as an internal control. The experiments were repeated three times using independent biological samples. Mean expression values and standard deviation are presented.

Mobility of the silencing effects is required for gene functional inference studies using VIGS. We checked the bleaching phenotypes in the *PfPDS*-*TRV2* infected *Physalis* thoroughly. In comparison with non-treated wild-type (Figure 2A) plants, the development of the bleaching phenotypes in VIGS treated plants features 2 characteristics. As the *PfPDS*-*TRV2* infected seedlings grew, the bleaching phenotype was also found to be present in newly developing organs (Figure 2B and C). This can be considered an advantageous characteristic of VIGS in *Physalis*. In comparison with wild type plants (Figure 2A, D, and F), the bleaching firstly appeared in the newly developed leaves of VIGS treated plants (Figure 2B), and then appeared sequentially on the flower bud, flower, and fruit (Figure 2C, E and G), suggesting that a local infection could induce a systemic effect.

When the seedlings were infected with *PfPDS*-*TRV2* and grown under the optimal conditions for VIGS, in comparison to wild-type ICS and berries (Figure 2H and I), the bleaching phenotype was observed in the ICS (Figure 2J and K) and berries (Figure 2L) in the *PfPDS*-*TRV2* plants. Unlike the yellow wild-type seeds (Figure 2M), some seeds from the bleached berries were completely bleached (Figure 2N), thus further substantiating our assertion that TRV-mediated gene silencing effects in *Physalis* are systemic and can last for an extended period of time, entailing all developmental stages.

These observations demonstrate that TRV-VIGS can be used for gene functional inference studies involving all developmental processes in *Physalis*. While this result is clearly useful and promising, we have to mention another characteristic of TRV-VIGS in *Physalis* that is likely disadvantageous. This problematic characteristic is that the bleaching phenotype was mosaic in nature. This type of bleaching could be seen on the leaves, calyx, corolla, ICS, and berries (Figure 2). Thus, the bleaching of organs has two different types: either mosaic or complete. What deserves to be mentioned is that mosaic ICS could completely cover the bleached berries and some bleached seeds developed in mosaic white berries. These results underscore the importance of careful phenotypic characterization when using VIGS.

### The Extent of Silencing of *PfPDS* Correlates to the Extent of Organ Bleaching

In order to evaluate the specificity of gene silencing in our VIGS experiments, we analyzed the causal relation between the bleaching phenotypes and the expression level of the endogenous *PfPDS*. In the *PfPDS*-*TRV2* plants, we collected unbleached and bleached organs including leaf, flower bud, mature flower, and berry of both the mosaic and the complete bleaching phenotypes. Total RNA from these organs were isolated and analyzed with qRT-PCR. Expression of the endogenous *PfPDS* was not altered in the unbleached organs in comparison with that of the wild-type. However, the expression level of *PfPDS* was knocked down to different degrees in the *PfPDS*-VIGS organs. The expression level of the un-silenced mRNA transcripts of *PfPDS* in mosaic type organs was higher than the expression observed in the completely bleached organs (Figure 2O), suggesting that the organ bleaching extent correlates with the extent of knockdown of *PfPDS* in the organs (in leaf *R* = 0.97, *p* = 0.031; in flower *R* = 1.00, *p* = 0.002; in ICS *R* = 0.96, *p* = 0.037; and in berry *R* = 0.98, *p* = 0.017). Further, this result provides molecular genetic evidence that *PfPDS*, which is an ortholog of *PDS* from other plants [21], [22], [30], is indeed involved in the carotenoid biosynthetic pathway in *Physalis*.

### Silencing *PfPDS* Has No Influence on the Developmental Quality of the Post-Floral Organs

The occurrence of few or no side-effects in a VIGS system is critically important for properly inferring the function of a gene. The possibility of various side-effects was evaluated in *PfPDS*-*TRV2* plants. As PDS is an enzyme in the carotenoid biosynthetic pathway, the bleaching of organs should represent the primary phenotypic variation observed once it is silenced. However, in a VIGS system using *PDS* as the reporter in tomato, seed quality was also affected [40]. Following on from this report, we checked for potential effects of *PfPDS* in alteration of the function of the *Physalis* reproductive system. The fruit setting rate, berry weight, and seed number per fully developed bleached fruit were the indicators that we used for the evaluation of reproductive function and fertility. 50 flowers of the wild-type, the mosaic, and the completely bleached phenotypic groups were randomly labeled, and the berries from these flowers were harvested at maturity. The mean fruit setting numbers for each group were 48, 47, and 49, respectively. Compared with the wild type (0.388±0.037 g), the mean berry weight of the mosaic and the completely bleached groups was 0.386±0.038 g (*p = *0.95) and 0.370±0.030 g (*p = *0.55), respectively. The mean seed number per berry of the wild-type, the mosaic, and the completely bleached groups was 151.0±35, 151±29 (*p = *1.00) and 148.0±4.0 (*p = *0.89), respectively (Figure S2A in File S1). Therefore, the self-fruit setting rate, berry weight, and the seed number per berry in *PfPDS*-VIGS had no statistic differences from those of the wild-type. In addition, we have reported previously that male fertility is not affected by silencing the *PfPDS* gene [13].

Premature seed germination was observed in tomato with *PDS* silenced via VIGS [40]. This was not observed for *Physalis*. We nevertheless compared the seed germination rates between wild-type and bleached seeds. We germinated 50 wild-type seeds and 50 completely bleached seeds under the optimal VIGS temperature (21–25°C). The germination rate was 98.0% in wild-type seeds, 96.0% in the bleached seeds and 97.6% in seeds of the bleached berries from *PfPDS*-VIGS (*p = *0.27; Figure S2B in File S1), implying that the *PfPDS*-VIGS seeds were well-developed.

Our results indicate that *PfPDS* has no role in either the quality or the functionality of the post-floral organs. We did not observe any other side-effects in our use of the TRV system in *Physalis*. As such, this system likely has board applicability for inferential studies of gene function.

### Non-Inheritance of *PfPDS*-TRV-VIGS Phenotypes

In our previous germination analyses, no seedlings featured bleaching phenotypes, suggesting that the TRV-VIGS effect was not heritable. In order to further verify this, 500 mature seeds from different *PfPDS*-*TRV2* fruits were analyzed. In total, 488 seeds were germinated and none of them became bleached (Figure S2B in File S1), thus confirming the non-heritability of the *PfPDS* silencing effect in the TRV-VIGS system in *Physalis*. However, under strictly controlled temperature conditions, viral vectors can remain in seeds for future generations, and silencing effects have been maintained for more than two years in *Nicotiana* [38]. Despite this report, the efficacy of VIGS is generally held to be transient.

### Applying the VIGS Strategy to Silence MADS-Box Regulatory Genes in *Physalis*

In order to substantiate the application of the TRV-VIGS technology in *Physalis*, we silenced the genes encoding the transcription factors. Two MADS-box genes, *MPF2* and *MPF3,* were down-regulated via RNAi interference approaches in our previous work, and the RNAi plants had specific phenotypic variation compared to the wild type [1], [13]. The specific fragments of *MPF2* and *MPF3* used for RNAi were here integrated into the TRV-system to create *MPF2-* and *MPF3*-VIGS constructs (Figure 1). These constructs were used to infect the leaves of *Physalis* seedlings [13]. Here we focused on comparing the silencing effects between the VIGS- and the RNAi- silenced plants.

#### *MPF2*-VIGS flowers phenocopy the *MPF2* -RNAi flowers

**Figure 3:**
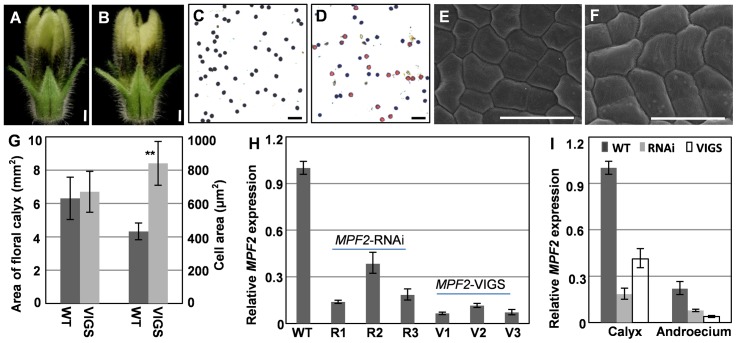
VIGS-mediated *MPF2* silencing phenocopies *MPF2*-RNAi. (**A**) An intact WT flower. (**B**) An intact *MPF2*-VIGS flower. Bars = 1 mm. (**C**) I_2_-KI stained pollen from WT. (**D**) I_2_-KI stained pollen from *MPF2*-VIGS. Active pollen is blue and sterile pollen is tawny. Bars = 100 µm. (**E**) Floral calyx epidermal cells of WT. (**F**) Floral calyx epidermal cells of *MPF2*-VIGS. Bars = 20 µm. (**G**) Size of calyx surface (gray column) and epidermal cells (white column) of the floral calyx in WT and *MPF2*-VIGS (“VIGS”). 20 cells and 20 calyces were analyzed for both WT and *MPF2*-VIGS samples. Mean values and standard deviation are presented. (**H**) Gene expression analysis of *MPF2*-RNAi and -VIGS. Expression of *MPF2* was compared between *MPF2*-RNAi flowers (R1–R3), *MPF2*-VIGS flowers (V1–V3) and wild-type (WT) *Physalis* via qRT-PCR analysis. The severe *MPF2* residual in VIGS was only 6% of that in the wild-type (WT), while in the RNAi the *MPF2* residual was 14% of that in the wild-type (WT). *PFACTIN* was used as an internal control. (**I**) *MPF2* expression was evaluated in two floral organs of VIGS flowers. Expression of *MPF2* in *MPF2*-RNAi (gray column), *MPF2*-VIGS (white column) was compared with that in the wild-type (WT, black column). The gene expression in the calyx of the WT was set as 1, and *PFACTIN* was used as an internal control. The experiments were repeated with three independent biological samples. Mean expression values and standard deviation are presented.

A successful *MPF2* infection had no obvious variation in mature flower surface phenotype (Figure 3B) as compared to the wild-type (Figure 3A). However, the pollen maturation of *MPF2*-VIGS, as revealed by I_2_-KI staining (50.8%±7.8%, Figure 3D), resembled that of the *MPF2*-RNAi plants [1] and was significantly lower than that of the wild-type (98.7%±0.9%, Figure 3C) plants. Total mRNA from flowers and two floral organs with decreased pollen maturation from the wild-type, *MPF2*-RNAi, and *MPF2*-VIGS plants was analyzed via qRT-PCR. The transcript abundance of *MPF2* in these mutated flowers and in each examined floral organ was efficiently knocked down in both cases (Figure 3H and I). Compared to the wild-type floral calyx (Figure 3E), knocking down *MPF2* did not alter calyx cell morphology (Figure 3F), but resulted in the development of significantly larger cells (*p*<0.001, Figure 3G). Therefore, the results obtained in the *MPF2*-VIGS analyses are consistent with the previous observations from earlier *MPF2*-RNAi experiments [1].

#### *MPF3*-VIGS flowers resemble the *MPF3* -RNAi flowers

**Figure 4:**
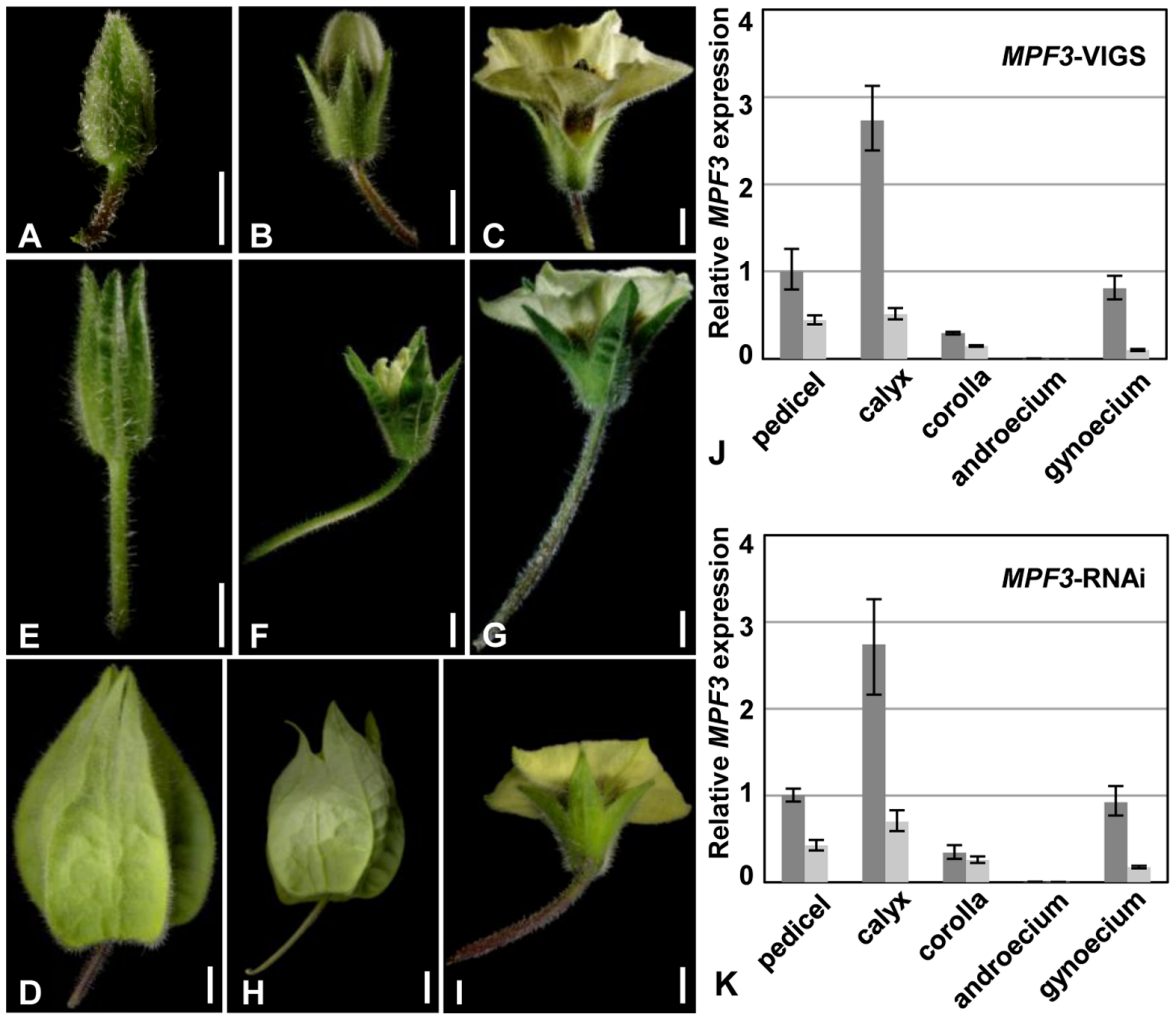
VIGS-mediated *MPF3* silencing phenocopies *MPF3*-RNAi. (**A**) An intact WT small flower bud. (**B**) An intact WT flower bud. (**C**) A WT flower. (**D**) A WT ICS. (**E**) An intact *MPF3*-VIGS small flower bud. (**F**) An intact *MPF3*-VIGS flower bud. (**G**) An *MPF3*-VIGS flower. (**H**) An *MPF3*-VIGS ICS. (**I**) An *MPF3*-RNAi flower. Bars = 1 mm in **A**, **B**, **C**, **E**, **F**, **G,** and **I**. Bars = 5 mm in **D** and **H**. (**J**) *MPF3* was silenced using a VIGS approach. *MPF3* expression was evaluated in five floral organs of VIGS flowers. (**K**) *MPF3* was silenced using an RNAi approach. *MPF3* expression was evaluated in five floral organs of RNAi flowers. Total RNA from the indicated mutated floral organs was subjected to qRT-PCR. Gene expression in pedicels of WT samples were set as 1, and *PFACTIN* was used as an internal control. The dark gray column stands for the gene expression in WT organs light gray column indicates the gene expression in the organs of the mutants, as indicated. The experiments were repeated with three independent biological samples. Mean expression values and standard deviation are presented.

Compared to the wild type (Figure 4A–D), knockdown of *MPF3* with the TRV-VIGS system caused formation of a leaf like calyx, an elongated pedicel, and a smaller, deformed ICS (Figure 4E–H). These phenotypes were also observed in RNAi studies (Figure 4I) [13]. Total mRNA from the floral organs of *MPF3-*VIGS and *MPF3 -*RNAi plants with dramatic phenotypic deviation from wild-type plants was extracted and analyzed with qRT-PCR. In each floral organ of both the *MPF3*-RNAi and the *MPF3*-VIGS plants, the expression of *MPF3* was significantly decreased (Figure 4J and K). Accordingly, in both cases, the pollen maturation is severely blocked [13]. Thus, both *MPF3-*VIGS and *MPF3*-RNAi result in similar phenotypic effects in flowers.

#### *MPF3* and *MPF2* regulate pedicel development and pedicel cell length

**Figure 5:**
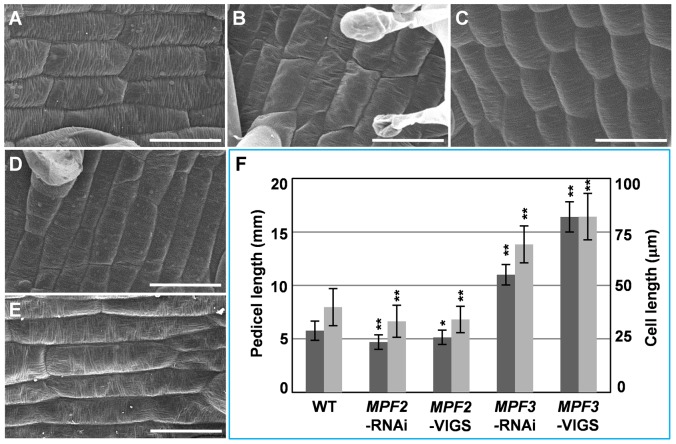
*MPF3* and *MPF2* regulate pedicel development and pedicel cell length. Pedicel cells from the WT (**A**), *MPF2*-RNAi (**B**), *MPF2*-VIGS (**C**), *MPF3*-RNAi (**D**), and *MPF3*-VIGS (**E**). Bars = 50 µm. (**F**) Quantification of the pedicel size (dark gray column) and the respective pedicel cells lengths (light gray column). The number of pedicels analyzed was 20 for each line above. The numbers of cells analyzed were 60 in WT, *MPF2*-RNAi and *MPF2*-VIGS, and 50 in *MPF3*-RNAi and *MPF3*-VIGS. Mean values and standard deviation are presented in both cases. Two-tailed *t*-test significance was given as follows: one star for *p*<0.05, and two stars for *p*<0.01.

The flowers of *MPF3* and *MPF2* downregulated mutants showed an altered pedicel length that we observed in our previous work, but they were not documented in detail [1], [13]. The flowers of these *MPF3* downregulated mutants showed an elongated pedicel (Figure 4E–I and 5F), while *MPF2* downregulated flowers had short pedicels (Figure 5F). These observations are consistent with that overexpressing *MPF2*-like cDNAs produced the elongated pedicels in transgenic *Arabidopsis* [4]. SEM analyses of pedicel cells from WT, *MPF2*-RNAi, *MPF2*-VIGS, *MPF3*-RNAi, and *MPF3*-VIGS flowers are presented in Figure 5A–E. Both pedicel size and pedicel cell lengths in these were quantified (Figure 5F). The pedicel sizes decreased by 18.5% in *MPF2*-RNAi plants compared to the wild type (*p*<0.001), and the pedicel cell lengths was decreased by about 16.6% compared to the wild type (*p*<0.001). The pedicel sizes of the *MPF2*-VIGS plants decreased by 10.7% compared with wild-type (*p = *0.013), and the pedicel cell lengths decreased by around 14.5% compared to the wild-type (*p*<0.001). The pedicel sizes of *MPF3*-RNAi plants increased by 80.8% compared with to the wild-type (*p*<0.001), and the pedicel cell length increased by around 73.7% compared to the wild-type pedicel cells (*p*<0.001). The pedicel sizes of *MPF3*-VIGS plants increased by 1.8 times compared with the wild type (*p*<0.001), and the pedicel cell lengths increased to about 1.1 times the size of the wild-type pedicel cells (*p*<0.001). Thus, these results suggested that the altered pedicel sizes were mainly caused by cell elongation and cell shortening, because the extent of variation of the pedicel size and its cell sizes was more or less equivalent among these knockdowns (*p = *0.67). Again, both *MPF3-*VIGS and *MPF3-*RNAi resulted in similar phenotypic effects in pedicel growth, the same held for both VIGS and RNAi of *MPF2*, implying that *MPF3* and *MPF2* may have adopted novel roles in pedicel growth in *Physalis*.

### VIGS Has Pronounced Advantages over RNAi Technology in *Physalis*

Very recently, it was shown that the TRV system did not give a robust and effective gene silencing effects in *Physalis philadelphica* [41]; however, we successfully applied the TRV-VIGS system in *Physalis floridana*. This might reflect a subtle difference in the host selection of TRV within the Solanaceae, even in *Physalis*. As with *PDS* orthologs encoding phytoene desaturases in various plants [21], [22], [24], [27], [31], [32], we showed that *PfPDS* functions in a conserved role in the carotenoid biosynthetic pathway in *Physalis*. Silencing *PfPDS* caused the bleaching of organs but did not affect the quality of the reproductive organs. This indicated the specificity and lack of developmentally deleterious side-effects for use of the TRV-VIGS system in *Physalis*. The essential components of an optimal VIGS procedure, including infiltration by the injection method and optimized temperature (21–25°C) were characterized for TRV-VIGS methods in *Physalis*. We previously demonstrated that *MPF2* and *MPF3* (MADS-box genes encoding transcription factors) specified floral calyx identity, controlled post-floral calyx inflation, and determined male fertility [1], [13]. Here, we further demonstrate the roles of *MPF2* and *MPF3* in pedicel development and growth. Silencing *MPF2* and *MPF3* using VIGS resulted in similar phenotypic variations as with those observed in experiments using an RNAi approach, indicating the applicability of the VIGS technique.

Three main advantages in practical aspects were found in the VIGS system against the RNAi technology. The TRV-VIGS features a high infection efficiency (more than 70%), shortens the required time for analysis to around 2–3 months for observation of phenotypic variation, and avoids the labor-cost tissue culture processes, while the RNAi in *Physalis* had a transformation efficiency of no more than 10%, and the length of time before phenotypes could be observed was approximately one year, and demands a large amount of tissue culture work in strict aseptic conditions [1], [12], [13]. Another obvious advantage of the TRV-VIGS system is the continuity of phenotypic variation in one infected plant (there are a series of knockdown degrees of target gene, there are different phenotypes), while at least 3 independent lines of the RNAi transgenic plants with a target gene downregulated are needed to evaluate phenotypic variations. Use of the TRV-VIGS system in *Physalis* will facilitate the elucidation of the molecular basis of the development and evolution of trait diversity, including ICS and berry size. TRV-VIGS has been successfully established in several Solanaceous species, like in *Petunia* [28], *Solanum* [22], [26], [29], [40], *Capsicum* [27], *Nicotiana* [23], [38] and *Physalis* [13]. The universality of this transient system of gene function inference will therefore play a role in revealing the functional evolution of some of the important genes within the Solanaceae.

## Conclusions

The local TRV-VIGS treatment can efficiently induce a systemic mutated phenotype in *Physalis floridana*. Compared to the RNAi technology, the TRV-VIGS system demands shorter time and lesser labor cost, and features higher infection efficiency and the continuity of phenotypic variation in one infected plant. And the VIGS-mediated gene knockdown plants phenocopy the RNAi transgenic plants. Besides the important roles in the development of floral calyx, fruiting calyx and male sterility, the MADS-box genes *MPF3* and *MPF2* are involved in the pedicel development in *Physalis*. The major contributions of the present work are to have established an optimal protocol for the TRV-medicated gene silencing in *Physalis floridana*, and to provide an efficient way to infer gene function in various developmental processes in *Physalis*, thus facilitating understanding of the genetic basis of the evolution and development of trait diversity within the Solanaceae.

## Supporting Information

File S1 **Figure S1, Figure S2 and Table S1.**
(PDF)
