# Supplementary material for: Efficient Gene Silencing Mediated by Tobacco Rattle Virus in an Emerging Model Plant Physalis
Source: PLoS One. 2014 Jan 14;9(1):e85534. doi: 10.1371/journal.pone.0085534 (PMC3891815; doi:10.1371/journal.pone.0085534)
Supplement: File S1 — Figure S1, Figure S2 and Table S1. (PDF) [file pone.0085534.s001.pdf]

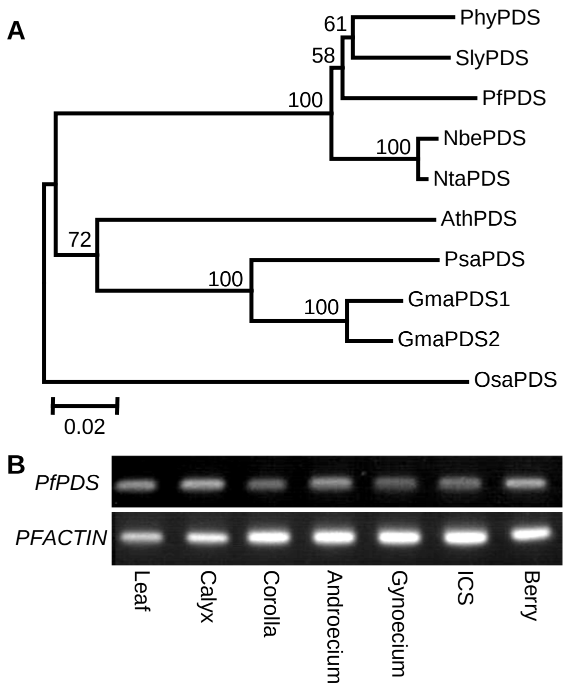

Figure S1. Characterization of *PfPDS* in *Physalis.*
(A) A Neighbor-Joining tree of the *PDS* genes. The accession numbers of the sequences are AY593974 (PhyPDS, *Petunia hybrida*), EF650011 (SlyPDS, *Solanum lycopersicum*), EU165355 (NbePDS, *Nicotiana benthamiana*), AJ616742 (NtaPDS, *Nicotiana tabacum*), AEE83394 (AthPDS, *Arabidopsis thaliana*), AJ621573 (PsaPDS, *Pisum sativum*), NM_001249840 (GmaPDS1, *Glycine max*), AK244738 (GmaPDS2, *Glycine max*), AF049356 (OsaPDS, *Oryza sativa*), and JX255734 (PfPDS, *Physalis floridana*). Bootstrap values of 1000 replicates are shown. (**B)** Expression of *PfPDS* in *P. floridana*. RT-PCR analysis was performed using total RNA from the different organs indicated. *PFACTIN* was used as an internal control. The experiments were performed with three biological replicates; typical, representative gel images are presented for each experimental result.

A

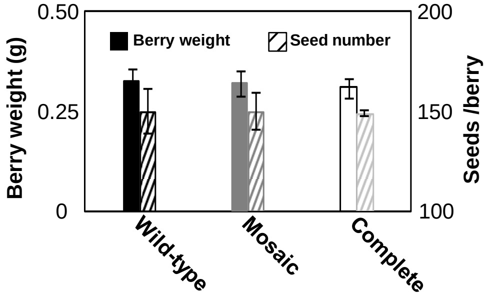

B

Seed resources	Germination rate	Seedling Phenotype
Wild-type seeds	98.0%	wild-type
The bleached seeds	96.0%	wild-type
Seeds from the bleached berries	97.6%	wild-type

Figure S2. Quantity of berries and seed characteristics from the *PfPDS*-TRV2 infected plants.
(A) Berry weight and seed number in the berries, with different degrees of bleaching indicated. (**B)** Seed germination rate and inheritance of the *PfPDS* silencing phenotype mediated by *PfPDS-TRV2*.

Table S1. Primer information used in the present work.

Usage	Gene name	Sequence
Real time RT-PCR	*MPF3*	F: 5’-GGA GAT GAA GCA AGG AGA AAT G-3’
		R: 5’-AAC TTC CCA AGC AAA GAT TA-3’
	MPF2	F: 5’-GTG CTG AGC TGA TGG AAG AA-3’
		R: 5’-CTT CAA CAA AAT CTT CCC AC-3’
	PfPDS	F: 5’-CCA GTG GAT ATT CTC AAG C-3’
		R: 5’-GAC ATG TCA GCA TAC ACA C-3’
	PFACTIN	F: 5’-AAC CGG AAT GGT GAA GGC TGG-3’
		R: 5’-CCA TAT CAT CCC AAT TGC TGA C-3’
RACE	*PfPDS*-3’UTR	F1: 5’-GAG TCA AAA GGT GGC CAA G-3’
		F2: 5’-GGA GAT GCT TTT GTG TTT GCC-3’
	PfPDS-5’UTR	R1: 5’-ATG ATC ATA TGT GTT CTT CAG-3’
		R2: 5’-CTG TCA AAC CAT ATA TGT AC-3’
VIGS	*PfPDS*	F: 5’-CTA CCA TGG GGC ACT CAA CTT TAT AAA CC-3’
		R: 5’-CTA GGA TCC CTT CAG TTT TCT GTC AAA CC-3’
	MPF3	F: 5’-CGC CAT GGT CAA GAA AGA ACC AAC TCA T-3’
		R: 5’-GCG GAT CCA GAT TTT ACG AGA ACA TAT T-3’
	MPF2	F: 5’-CGC CAT GGC CAG CAT GAA GGA TAT CCT T-3’
		R: 5’-GCG GAT CCT GCT AGC TGA GTG GTA GCC C-3’
